# Application of Different Animal Fats as Solvents to Extract Carotenoids and Capsaicinoids from Sichuan Chili

**DOI:** 10.3390/foods13101478

**Published:** 2024-05-10

**Authors:** Bingyu Zheng, Yida Wu, Yong Wang, Ying Li

**Affiliations:** Guangdong International Joint Research Center for Oilseeds Biorefinery, Nutrition and Safety, Department of Food Science and Engineering, Jinan University, Guangzhou 510632, Chinatwyong@jnu.edu.cn (Y.W.)

**Keywords:** animal fats, green extraction, carotenoids, capsaicinoids, Sichuan spicy hotpot oil

## Abstract

Inspired by the proved dissolving power of vegetable oils for non-polar and low-polar natural compounds, animal fats with triglycerides as the major components were investigated as food-grade solvents in this study for the simultaneous extraction of carotenoids and capsaicinoids from Sichuan chili. The dissolving power of lard, beef tallow, chicken fat and basa fish oil in the extraction of er jing tiao chili was firstly compared, where animal oils with worse extraction ratios for carotenoids (0.79 mg/g in average) performed better for the extraction of capsaicinoids (0.65 mg/g in average). Furthermore, the solvent effect of animal fats on the oleo-extracts was evaluated in terms of fatty acid composition, oil quality indexes, crystal polymorphism, melting and crystallization behaviors, where no significant differences were observed between animal fats before and after extraction. The oxidative stability of animal fats could be 1.02- up to 2.73-fold enhanced after extraction and the pungency degree could reach the same spicy level as commercial hotpot oil. In addition, the Hansen solubility parameters of solvents and solutes were predicted for further theoretical miscibility study, which helps to make a better comprehension of the dissolving mechanism behind such oleo-extraction. Overall, animal fats demonstrated their considerable solvent power for extracting carotenoids and capsaicinoids simultaneously from Sichuan chili, which showed significant potential for developing a novel Sichuan spicy hotpot oil with enhanced flavor and stability.

## 1. Introduction

**Figure 1:**
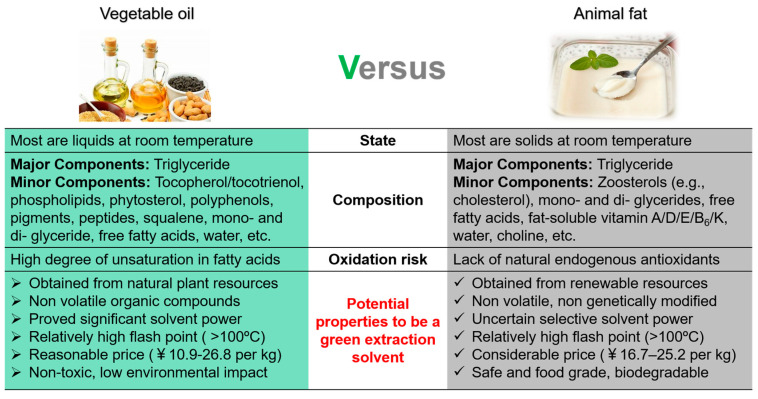
Comparison of basic properties between vegetable oils and animal fats.

Hotpot as an important part of Chinese food culture has a history of more than 2000 years [1], where the spicy hotpot that originated from the Sichuan and Chongqing areas has become more popular worldwide because of its unique flavor and growing consumer acceptability [2,3]. Animal fats and vegetable oil are the two main types of hotpot oil on the market. The former are generally solid at room temperature, which can be melted into the hotpot system with other food ingredients to improve flavor and taste. Beef tallow is the most frequently used for hotpot oil among animal fats [4]. Compared to animal fats, vegetable oil is plant-based and liquid at room temperature, which has moderate flavor without greasy feeling [5]. In addition to the sensory quality, there are other differences between animal fats and vegetable oil (Figure 1), where the most important one is the chemical composition. Although vegetable oil is recognized as healthier than animal fats due to its high degree of unsaturation in fatty acids, the major component in both animal fats and vegetable oil is still triglycerides (TAGs). Moreover, a rich variety of minor components like tocopherols, phytosterols and polyphenols are available in vegetable oils, which are rarely found in animal fats [6]. These endogenous natural compounds not only play a pivotal role in preventing oxidative deterioration in lipids, but can also act as surfactants for enhancing the dissolution of more polar compounds in oils [7]. Hence, although animal fats are susceptible to lipid oxidation during thermal processing due to fatty acid composition and lack of these minor nutrients [8], the overall quality including oxidative stability and flavor might be improved based on their dissolving power.

Until now, synthetic antioxidants such as tert-butylhydroquinone (TBHQ) have still been widely used to improve the oxidative stability in oil and fat products, though their adverse effects on human health have been gradually evidenced [9]. Therefore, industrial and consumer demand for natural antioxidants has grown quickly due to stricter standards and tighter regulation worldwide. The effect of green tea extract on the quality and shelf life of animal fats was investigated, where green tea bioflavonoids in an amount of 0.01% of fat weight showed a similar effect to 0.02% BHT without any negative effect on fat properties [10]. Rosemary extract also showed its best antioxidant effect below 130 °C [11]. The rancidity development in poultry fats could be significantly reduced by the addition of α-tocopherol and citric acid in a concentration of 0.1% [12]. Moreover, the addition of burdock extract at a concentration of 0.01% could significantly reduce the oxidation process in lard and goose fat heated at different temperatures [13]. In addition, natural antioxidants extracted from olive mill wastewater proved their effectiveness and safety in the oxidative stabilization of lard [14]. Therefore, it can be seen that animal fats may have considerable dissolving capacities for natural antioxidants of different polarities like vegetable oils [15]. The optimized oleo-extraction of natural compounds could be proposed as an efficient way of improving both oxidative stability and functionality of original fats. Hence, according to the ideal properties of green solvents and green extraction principles [16], it is meaningful to investigate the solvent power of representative animal fats with a similar TAG composition to vegetable oils, which to date has been rarely studied.

Chili pepper is a natural plant source rich in carotenoids consisting of capsanthin, capsorubin, zeaxanthin, β-cryptoxanthin and β-carotene [17], which are important natural compounds beneficial for human health. The conventional way of making spicy hotpot is to heat beef tallow until melted first and then to mix Sichuan chili pepper and other spices for stir-frying and cooking afterwards, where er jing tiao chili is indispensable to authentic Sichuan cuisine as the seasoning. The notable antioxidative effect of carotenoids and capsaicinoids was also demonstrated in previous studies [18,19,20]. Thus, this endemic capsicum variety was chosen as the raw material in this study. For the sake of selective extraction and functional application with animal fats in future, it is important to previously understand their dissolving capacities. Hence, four different animal fats as food-grade solvents were first experimentally investigated to simultaneously extract carotenoids and capsaicinoids from er jing tiao chili, which was intended to make a novel Sichuan hotpot oil with enhanced flavor, stability and nutritional value. The overall performance of these solvents before and after extraction was evaluated by the comparison of fatty acid composition, acid value, total oxidation value, oxidative stability, melting and crystallization behaviors, and crystal polymorphism as well. In addition, the Hansen solubility parameters (HSPs) were also introduced for better understanding the dissolving mechanism from the theoretical point of view.

## 2. Materials and Methods

### 2.1. Materials

Dried er jing tiao chili (*Capsicum annuum* L.) was provided by Youangonggeng E-commerce Co., Ltd. (Chongqing, China). Animal fats including chicken fat, lard, beef tallow and basa fish oil were purchased from Qiu Dasao E-commerce Co., Ltd. in Wuhe county (Bengbu, China), Xiangshui Agriculture Development Co., Ltd. (Yueyang, China), Hangjia Biotechnology Co., Ltd. (Deyang, China) and Honoroad Co., Ltd. (Chongqing, China), respectively. Spicy hotpot oil from the most popular brand in China was obtained from the local supermarket.

Capsaicin (97% purity), dihydrocapsaicin (90% purity) and capsanthin (BR grade) were obtained from Maclin Biotechnology Co., Ltd. and Aladdin Biotechnology Co., Ltd. in Shanghai, China, and the Jim-bio Co., Ltd. in Beijing, China, respectively. AR grade of p-methoxyaniline and thymolphthalein were also obtained from Maclin Biotechnology Co., Ltd. and Aladdin Biotechnology Co., Ltd. in Shanghai, China, respectively. High performance liquid chromatography grade methanol and acetonitrile were supplied by Mirida Technology Co., Ltd.in Beijing, China. Soluble starch, glacial acetic acid and potassium iodide, bought from Damao chemical reagent factory (Tianjin, China), were of AR grade. All other reagents of AR grade were provided from the local chemical reagent factory in Guangdong, China, including acetone, isopropanol, trichloromethane, isooctane, n-hexane, potassium hydroxide, boron trifluoride methanol complex solution and standard solution of sodium thiosulfate (0.1 mol/L).

### 2.2. Extraction Using Animal Fats as Solvents

Er jing tiao chili was ground into powder using a 60-mesh screen. Four different animal fats were used as solvents with a material ratio (40%, *w*/*v*) in the magnetic stirring extraction at 50 °C for 1.5 h, which was then filtered for the following analyses as compared to commercial hotpot oil. The total content of carotenoids, capsanthin and capsaicinoids in er jing tiao chili powder was thoroughly extracted in a traditional way using hexane, acetone and methanol–water solution (80/20, *v*/*v*) as solvents, respectively. The extraction rate of animal fats for total carotenoid content, capsanthin and capsaicinoids was calculated by the general formula as follows,

Extraction rate (%) = X_1_/X_2_ × 100 (1)

where X_1_ is the content of carotenoids, capsanthin or capsaicinoids extracted by animal fats after extraction (μg/g or mg/g) and X_2_ is the total content of carotenoids, capsanthin or capsaicinoids in er jing tiao chili samples (μg/g or mg/g).

### 2.3. Determination of Total Carotenoid Content

The determination of total carotenoid content was slightly modified based on the previous method [11]. The mixture of all samples (0.5 g) and acetone (75%, 5 mL) was stirred with 15 mL of a hexane-acetone-ethanol mixture (2:1:1) for 0.5 h, which was then mixed with 2.5 mL of distilled water for 5 min and centrifuged for 15 min at 3000 rpm. The extraction procedure was repeated with 15 mL of hexane and the upper layer of hexane was quantitatively removed and combined for determining total carotenoid content, which absorbance was measured by an UV-5500PC ultraviolet spectrophotometer (Metash Instruments, Shanghai, China) at 450 nm with hexane as the blank. The content of total carotenoids was calculated as described in the following Equation (2):

(2) $$Total\;carotenoid\;content\left(\frac{\mu g}{g}\right)=\frac{A\times V\times 10000}{\varepsilon \times P}$$

where A is the absorbance at 450 nm; V is the total volume of hexane layer (mL); ε($A\frac{1\%}{1\;cm}$) is the extinction coefficient of mixture of carotenoids in hexane (2500 dL/g·cm) and P is weight (g) of sample.

The capsanthin standard solution was prepared in acetone by gradient dilution (50.00, 33.33, 25.00, 16.67 and 12.50 μg/mL), which was then measured by the same UV-5500PC spectrophotometer at 483 nm in order to make the standard curve of capsanthin (absorbance = 84.015 × concentration(μg/mL) − 4.2227, R^2^ = 0.9912). Capsanthin in the raw materials was extracted several times by acetone until they become colorless. Samples extracted by animal fats (0.02 g) were dissolved in 10 mL of acetone, which capsanthin content could be directly determined at 483 nm according to the standard curve.

### 2.4. Determination of Capsaicinoids and Pungency Degree

Capsaicinoids in animal fats after extraction were analyzed according to Fang et al.’s method [21]. At first, standard solutions of capsaicin (0.025, 0.05, 0.1, 0.2 and 0.25 mg/mL) and dihydrocapsaicin (0.02, 0.04, 0.08, 0.16 and 0.2 mg/mL) were separately prepared in methanol by gradient dilution, which were used to make standard curves based on the results of the chromatographic peak areas.

Samples of 0.5 g were mixed with 5 mL of methanol–water solution (80/20, *v*/*v*) and sonicated at 50 °C in water bath for 20 min. The supernatants were removed to the tube after centrifugation, which was mixed with 5 mL of methanol–water solution (80/20, *v*/*v*) again, then vortexed and centrifuged. The supernatants were combined and frozen at −20 °C for 5 h, and then filtered to have the clear solution. Samples were filtered through a 0.22 μm nylon syringe after centrifugation, which were then injected into a LC-20A HPLC system (Shimadzu, Kyoto, Japan) using a C18-A column (250 mm × 4.6 mm × 5 μm) with methanol–water solution (70/30, *v*/*v*) as the mobile phase. The injection volume was 10 μL and the flow rate was 0.5 mg/mL. The detection wavelength was 280 nm and the column temperature was 25 °C. The content of capsaicin and dihydrocapsaicin in animal fats could be calculated according to the corresponding standard curve. The content of capsaicin and dihydrocapsaicin in the chili powder was extracted for 1.5 h using methanol–water solution (80/20, *v*/*v*).

The pungency degree was calculated according to following equations in Chongqing local standard (DB 50/T 870-2018) [22].

(3) $$X=\frac{\rho \times V}{1000m}$$

where *X* is the content of capsaicin and dihydrocapsaicin in samples (g/kg); *ρ* is the mass concentration of capsaicin and dihydrocapsaicin in samples (mg/L); *V* is the constant volume of samples (mL) and *m* is the mass of samples (g).

(4) $$W=\frac{X_{1}+X_{2}}{0.9}$$

where *W* is the total content of capsaicinoids in samples (g/kg); *X*_1_ is the content of capsaicin in samples (g/kg) and *X*_2_ is the content of dihydrocapsaicin in samples (mg/L).

(5) $$Pungency\;degree\left({}^{\circ}\right)=10.369\times \ln\left(W\right)+62.264$$

The grading of pungency degree for the spicy hotpot base was divided into 6 levels according to the following range: 9–29° (slightly spicy), 30–39° (low spicy), 40–49° (medium spicy), 50–59° (high spicy), 60–69° (extra spicy) and ≥70° (super spicy).

### 2.5. Fatty Acid Composition Analysis

According to the AOCS Official Method Ch 3-91 [23], the fatty acid composition of animal fats before and after extraction was determined after transmethylation to fatty acid methyl esters (FAMEs). Gas chromatography (GC7820A; Agilent, Wilmington, DE, USA) equipped with a flame ionization detector (FID) was performed by a CP-sil88 capillary column (100 m × 0.25 mm × 0.2 mm) using nitrogen as the carrier gas under a constant flow pressure of 30.8 psi. About 0.8 μL of sample was injected with a split ratio of 40:1. The temperature for both injector and detector was 260 °C. The oven temperature was programmed to maintain at 120 °C for 3 min, then heated to 175 °C at 8 °C/min and held for 18 min. All data were collected with Agilent EZChrom Elite 3.2.0 software. The fatty acid composition was quantified as relative percentages of the total fatty acids after identification by comparison with 37 FAME standards (Supelco, Bellefonte, PA, USA).

### 2.6. Acid Value and Total Oxidation Value (TOTOX)

The determination of the acid value, peroxide value (PV) and *p*-anisidine value (*p*-AV) was performed according to AOCS Cd 3d-63 [24], AOCS Cd 8b-90 [25] and AOCS Cd 18-90 [26], respectively. The TOTOX value was calculated as the sum of 2-fold PV (meq O_2_/kg) and *p*-AV [27].

### 2.7. Melting and Crystallization Behaviors

Melting and crystallization behaviors were analyzed with a Q2000 DSC differential scanning calorimeter (TA Instruments, New Castle, DE, USA) [26]. Nearly 5 mg of animal fat samples were weighed and sealed in aluminum pan with an empty pan as the reference. Samples were first cooled down to −60 °C at 10 °C/min, held for 2 min, and then increased to 60 °C at the same rate for obtaining the melting curve. This temperature was kept for 5 min to destroy the crystal history and then reduced to −60 °C at 10 °C/min to obtain the crystallization curve.

### 2.8. X-ray Diffraction (XRD) Measurement

Polymorphic forms in animal fat samples were analyzed using a Bruker D2 PHASER X-ray diffractometer (Bruker AXS GmbH, Karlsruhe, Germany) with Cu–Kα radiation (wavelength of 1.54056 Å) and Ni filter. Samples were scanned from 5 to 30° at 2°/min and measured using 40 kV and 40 mA. Voltage and electricity were set as 40 kV and 40 mA, respectively [28]. Polymorphic forms were distinguished by short spacing values based on their XRD patterns: short spacing at 4.15 Å was for α crystal form and β form corresponded to 4.6 Å, short spacing values at approximately 3.8 and 4.2 Å were characteristic of the β′ polymorph.

### 2.9. Micromorphological Observation

Microstructural analysis of the animal fat crystal particles was captured using PLM polarized light microscopy (Leica DM2700P; Wetzlar, Germany) equipped with a Nikon D3300 digital camera (Nikon Corporation, Tokyo, Japan). All samples were heated at 50 °C in the water bath for 15 min to destroy the crystal memory. A drop of the molten sample was then transferred to a preheated glass slide, and covered with a preheated cover slip to make sure that the drop was homogeneously dispersed without bubbles formed. Samples were cooled in the self-assembly thermostat to crystallize at room temperature. The photomicrograph was taken at 20× magnification.

### 2.10. Oxidative Stability

The oxidative stability of animal fat samples was determined by measuring the oxidation induction time using the Rancimat method [29]. All measurements were conducted at 110 °C, where 3 g of oil sample was placed in the vessel of Metrohm-743 Rancimat lipid oxidation stability tester (Hongkong, China) and the air rate was set to 20 L/h.

### 2.11. Theoretical Solubility of Capsanthin and Capsaicinoids in Animal Fats

The theoretical miscibility between animal fat solvents and solutes (capsanthin and capsaicinoids) extracted was predicted by their Hansen solubility parameters (HSP), which could provide a rapid and useful way to characterize solute–solvent interactions [30]. HSP extends the simple Hilderbrand’s total cohesive energy density (δ_total_^2^), which is approximately the sum of the energy densities required to overcome atomic dispersion forces (δ_d_^2^), molecular polar forces arising from dipole moments (δ_p_^2^) and intermolecular hydrogen bonds (δ_h_^2^), as given in the following equation:

δ_total_^2^ = δ_d_^2^ + δ_p_^2^ + δ_h_^2^ (6)

where δ_total_ is the Hansen total solubility parameter, including three solubility parameters in terms of dispersion (δ_d_), polar (δ_p_) and hydrogen-bonding (δ_h_).

Generally, the more similar the two δ_total_ are, the greater the affinity between solutes and solvents. Therefore, the HSP of solvents and solutes could be investigated through their chemical structural transformation into simplified molecular input line entry syntax (SMILES) notations. These solubility parameters could be further modeled to their two- or three-dimensional HSP representations for better visualizing the solute/solvent interaction in HSPiP (Version 5.2.06, Copenhagen, Denmark).

### 2.12. Statistical Analysis

All experiments were repeated in triplicate and the data were expressed as means ± standard deviations. The statistical analysis of variance (ANOVA) was determined by the SPSS Statistics 16.0 software (IBM Corp., Armonk, NY, USA) using Tukey’s test with a significant difference at 95% level between samples. Significant differences (*p* < 0.05 or *p* < 0.01) showed by each sample tested were labeled as different superscript letters.

## 3. Results and Discussion

### 3.1. The Effect of Animal Fats as Solvents on the Extraction of Carotenoids and Capsanthin

**Figure 2:**
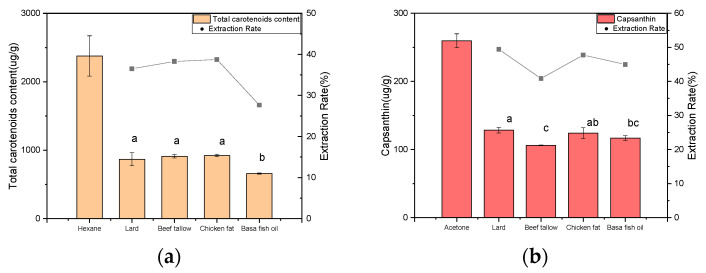
The effect of animal fats as solvents on the extraction of (**a**) carotenoids and (**b**) capsanthin. Different lowercase letters indicate that the data are statistically different (*p* < 0.05).

It is reported that the content of carotenoids depends on the variety of capsicum; some species had a total carotenoid content ranging from 1 to 6–8 mg/g, where the β-carotene content ranged from 0.045 to 0.85 mg/g [31]. The total content of carotenoids and capsanthin in er jing tiao chili extracted by hexane and acetone was 2.73 mg/g and 0.26 mg/g, respectively. As illustrated in Figure 2, the extraction rate of total carotenoid content by animal fats ranged from 27% to 39%, where no significant difference was found with the exception of basa fish oils. Civan and Kumcuoglu [20] found that the total content of carotenoids in olive oil extracts from the pulp of hot pepper paste was 1–2.3 mg/g with the assistance of ultrasound, which was much higher than the animal fats (0.66–0.92 mg/g) provided in this study. However, the average total carotenoid content in animal fats (0.79 mg/g) after chili (*Capsicum annuum* L.) extraction was much higher than other vegetable oils (0.23–0.26 mg/g) like corn, sunflower and safflower [32]. Capsanthin is a unique lipophilic red carotenoid in the chili fruits [33], which accounted for nearly 15% of the total carotenoid content of er jing tiao chili. It was interesting to notice that the extraction rate of all animal fats for capsanthin was higher than 40%, which was not directly proportional to the extraction rate for total carotenoid content. The highest extraction ratio for carotenoids and capsanthin was obtained by chicken fat and lard, respectively. This might be due to the degradation and isomerization of carotenoids caused by heat, light and oxygen effects, resulting in the loss of color and incomplete extraction of carotenoids [34,35]. Moreover, the content of total carotenoids (339.34 ± 1.53 μg/g) and capsanthin (27.11 ± 0.23 μg/g) in commercial spicy hotpot oil is much lower than animal fats after extraction. From this, it can be concluded that animal fats could have a noteworthy extraction efficiency for carotenoids, where the chain length and composition change in fatty acids seems to have no significant influence on the dissolving capacity of animal fats.

### 3.2. The Effect of Animal Fats as Solvents on the Extraction of Capsaicinoids

**Figure 3:**
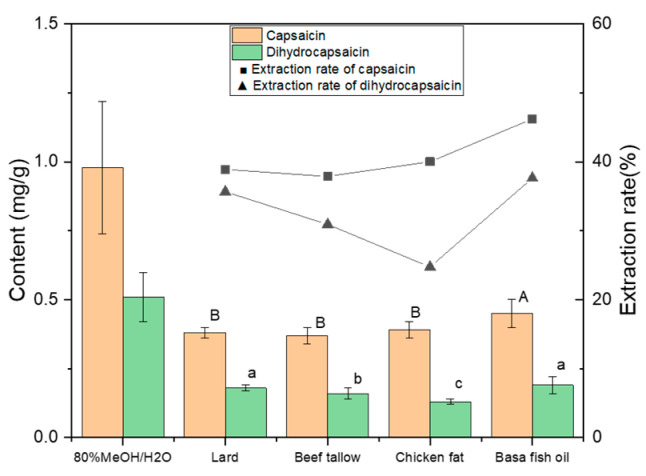
The effect of animal fats as solvents on the extraction of capsaicinoids. Different uppercase and lowercase letters indicate that the data are statistically different (*p* < 0.05) for capsaicin and dihydrocapsaicin, respectively.

The content of capsaicin and dihydrocapsaicin extracted in animal fats was also not directly proportional (Figure 3). Compared to aqueous methanolic extracts, basa fish oil showed the highest extraction rate for both capsaicin (46.25%) and dihydrocapsaicin (37.53%). However, its performance on total carotenoid extraction was the worst (27%). Chicken fat presented the second highest extraction rate for capsaicin (38.99%) but the lowest extraction rate for dihydrocapsaicin (24.66%), resulting in the lowest capsaicinoid content. This may be attributed to the relatively poor solubility of less polar dihydrocapsaicin with one more saturated bond in more polar chicken fat with a higher content of myristic acid [36]. Overall, these four animal fats performed better than olive oil in the extraction of capsaicinoids [37]. Moreover, the pungency degree of animal fats after extraction was calculated based on their total capsaicinoid contents. As compared to commercial hotpot oil with a pungency degree of 66.76° (extra spicy), only lard and basa fish oil could reach the same pungency level due to their higher capsaicinoid content extracted (Table 1), which might be used as alternatives in the future. According to Figure 2 and Figure 3, it is interesting to notice that animal fats (e.g., chicken fat) with a lower extraction rate of capsaicinoids have a higher extraction rate of carotenoids, and vice versa. Hence, considering the extraction ratio for both carotenoids and capsaicinoids, lard might be a compromise for developing a novel spicy hotpot oil.

### 3.3. Physicochemical Properties of Animal Fats before and after Extraction

#### 3.3.1. Fatty Acid Composition

The fatty acid composition in commercial hotpot oil is oleic acid (C18:1, 53.45 ± 0.16), stearic acid (C18:0, 22.69 ± 0.06), palmitic acid (C16:0, 10.88 ± 0.11), linoleic acid (C18:2, 7.19 ± 0.01) and palmitoleic acid (C16:1, 4.01 ± 0.02). According to the changes in the fatty acid composition of animal fats before and after extraction in Table 2, the major fatty acids in lard, beef tallow and basa fish oil were the same as those in commercial hotpot oil, while those in chicken fat were palmitic acid, oleic acid and linoleic acid. No significant difference was found for the fatty acid composition of chicken fat before and after extraction while the three other animal fats showed various degrees of significant changes in their fatty acid compositions. For lard and beef tallow, linoleic acid significantly increased after extraction while the content of other fatty acids reduced, especially for oleic acid in beef tallow. The same changing trend was found in the fatty acid composition of basa fish oil after extraction, where the unsaturation degree rose with the decline in all saturated fatty acids. On one hand, this may be due to the fact that the oxidation and dehydrogenation of saturated fatty acids during extraction at 50 °C could be converted into unsaturated fatty acids. On the other hand, linoleic acid as the predominant fatty acid in chili could be dissolved in animal fats during oleo-extraction [38], resulting in the increase in the unsaturation degree. On the whole, using animal fats as extraction solvents showed little effect on their fatty acid compositions, or even could facilitate the increment in unsaturation degree in animal fats.

#### 3.3.2. Oil Quality Indexes

As can be seen in Table 3, the acid value of animal fats after extraction was significantly increased compared to the original ones [39], which depends on the various extraction efficiency of animal fats. However, these increased acid values are still within limits according to the national standard for edible oil [40] and for the provincial standard for hotpot oil [41]. The peroxide value and *p*-anisidine value are generally used to reflect the degree of oxidation corresponding to primary and secondary oxidation products in oils and fats, respectively. Interestingly, both of these values in all animal fats after extraction were significantly reduced to a low level, leading to small TOTOX values as well. This might be due to the antioxidant effect of capsaicinoids, especially for the prevention of lipid hydroperoxide formation [42,43]. Regarding the oil quality indexes above, oleo-extraction could help animal fats to enrich bioactive components such as carotenoids and capsaicinoids, so that the oxidative stability of such animal fats could be improved.

#### 3.3.3. Oxidative Stability

The Rancimat method has usually been used to measure the oxidative stability of animal fats by detecting volatile acids formed during their oxidation [44]. Under the same Rancimat conditions, the longer induction period indicates a higher oxidative stability. Previous studies have evaluated the antioxidant potential of alcoholic extracts from red chili and the addition of red chili pepper powder to vegetable oils was proved to be effective in improving their stability during one year of shelf-life [45]. As presented in Table 3, the oxidation induction time (OIT) of most animal fats was significantly increased after extraction, which could be probably attributed to bioactive compounds extracted from er jing tiao chili. Regardless of extraction, the oxidative stability of chicken fat was always the lowest among animal fats because of its shortest OIT. The increase rate of OIT for lard was 272.6% after extraction, followed by chicken fat (168.5%) and beef tallow (101.9%). The oxidative stability of basa fish oil with the longest OIT before extraction showed a decline rate of 10.9% after extraction, which is inconsistent with the change in its TOTOX value. This might be associated with its increased unsaturation degree after extraction. Although OIT from the Rancimat method is a common way of determining the shelf life of oils and fats, it could not correspond to the actual storage at room temperature due to its different definition from that of shelf life [46].

#### 3.3.4. Melting and Crystallization Behavior

**Figure 4:**
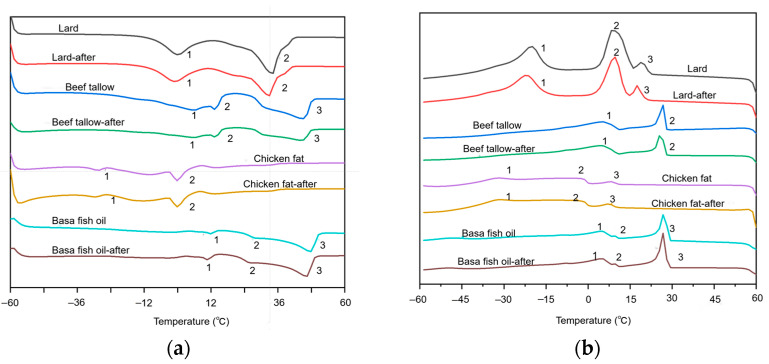
Melting and crystallization behaviors of animal fats before and after extraction: (**a**) melting behaviors; (**b**) crystallization behaviors.

The melting and crystallization properties are depicted by DSC thermograms, where various characteristic endothermic and exothermic peaks in the melting and crystallization curves of animal fats resulting from their different fatty acid compositions are illustrated in Figure 4 [47]. Animal fats with a higher saturation level have higher melting points and the enthalpy of fatty acids is affected by the chain length, the number of double bonds and the arrangement [48,49]. During the melting process, two endothermic peaks were observed for both lard and chicken fat while three peaks were found for beef tallow and basa fish oil. Chicken fat with a higher unsaturation degree represented two small endothermic peaks due to the lack of high melting point TAG molecules [50,51,52]. Basa fish oil with the highest melting point and enthalpy reflected its high thermal stability due to the presence of higher melting point saturated TAG molecular species [53]. It is noteworthy that the melting curve of animal fats before and after extraction was slightly changed. A similar phenomenon was also found for crystallization curves, indicating that extraction had no significant effect on the thermal behaviors of animal fats though they were enriched with carotenoids and capsaicinoids. Lard with a broad peak signified that various crystal cores were present in the crystal. On the contrary, a sharp peak signified that the triglyceride type during crystallization was single and the nucleation time was roughly the same [54]. The change in peak shape illustrated the change in polymorphism and the change in melting peak from 5 to 60 min was attributed to the polymorphic transition of α to β′ or β [55]. Furthermore, the position of the last exothermic peak of beef tallow and basa fish oil was similar, indicating that both two animal fats contained high melting constituents, resulting in the increased crystallization peak temperature and the enthalpy of heat release [56]. In addition, there is no obvious thermal transition in the crystallization curve of chicken fat, where a small crystallization peak was found near 10 °C.

#### 3.3.5. Crystal Polymorphism and Micromorphology

**Figure 5:**
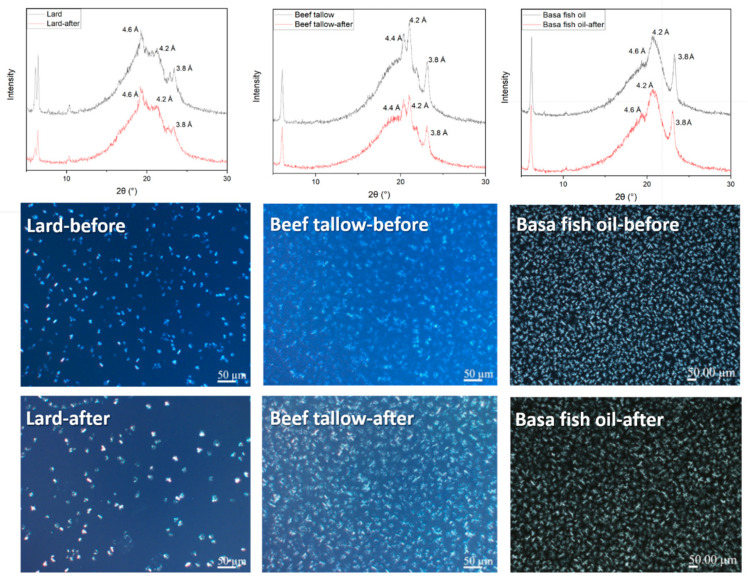
Crystal polymorphism and micromorphology of animal fats before and after extraction.

Generally, there are three major typical polymorphic forms in fats and oils including α, β′ and β crystal forms, among which α is unstable and easy to transform into other two types. As presented in Figure 5, the polymorphic form of chicken fat in liquid form at room temperature could not be detected in this work. Lard exhibited a relatively strong diffraction peak at 4.6 Å for β form and two weak diffraction peaks at 3.8 Å and 4.2 Å for β′ form, indicating that lard is a typical β form fat accompanying with few β′ forms. However, β′ crystal forms at 3.8 and 4.2 Å were dominant in beef tallow and a new diffraction peak at 4.4 Å was observed corresponding to the presence of the β crystal form. Basa fish oil had a similar diffractogram to that of beef tallow, which also had two strong diffraction peaks at 3.8 Å and 4.2 Å for the β′ form. The β′ form is often associated with asymmetric triglycerides while the β form is often related to symmetric triglycerides. It is worth mentioning that no new diffraction peaks appeared in animal fats before and after extraction, indicating that there were no significant changes in the arrangement of fatty acids in the TAG molecules [57].

PLM was applied to further verify the XRD results with a better visualization of the microstructure of fat crystal networks shown in Figure 5. The PLM images clearly showed that there were no transformations in the morphology of crystallized samples before and after extraction. Radiated needle-like crystals were mainly found in beef tallow and basa fish oil, which were clustered to a denser network structure after extraction. The microstructure of crystal networks in the lard was granular crystals, which were also clustered after extraction to become large in size but with a reduced compactness. The PLM results had good consistency with the XRD patterns, which demonstrated again the insignificant effect of oleo-extraction on the transformation of crystal polymorphism and microstructure in animal fats.

### 3.4. Theoretical Miscibility of Carotenoids and Capsaicinoids with Animal Fats

The solubility of carotenoids and capsaicinoids in animal fats could be theoretically predicted using their Hansen solubility parameters based on their chemical structures. Since target solutes and animal fat solvents are classified as non-standard molecules in the embedded HSP database, they required to be predicted in an accurate and efficient way using the Yamamoto-Molecular Break (Y-MB) method, which could break SMILES into corresponding functional groups for further HSP estimations. For instance, the chemical structure of capsanthin as a typical carotenoid in chili could be transformed to its SMILES notions as CC1=C(C(CC(C1)O)(C)C)C=CC(=CC=CC(=CC=CC=C(C)C=CC=C(C)C=CC(=O)C2(CC(CC2(C)C)O)C)C)C for calculating HSP directly by the Y-MB method. However, the current HSPiP version could not distinguish the isomer of molecules, so that both canonical and isomeric SMILES for capsanthin would have the same HSP at the moment. Furthermore, all configuration possibilities of fatty acids on the TAG of animal fats were considered and the average HSP of animal fats, carotenoids and capsaicinoids was used for the theoretical miscibility prediction. The HSP of all solvents and solutes were calculated and used in plotting the 2D HSP graph with a relative energy difference (RED) number as the criterion for evaluating theoretical miscibility between solvents and solutes.

**Figure 6:**
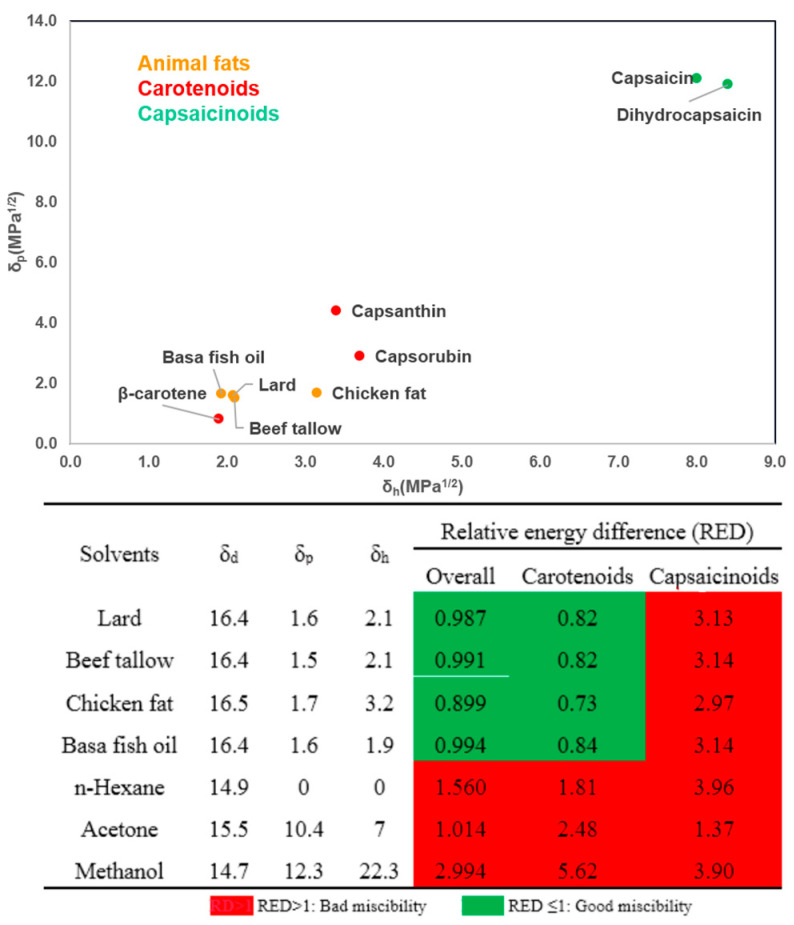
Theoretical miscibility between solutes (carotenoids and capsaicinoids) and solvents (animal fats and classic petroleum-based volatile solvents) based on the relative energy difference (RED) values predicted by their Hansen solubility parameters.

RED is the HSP distance between the given solvent and the reference value, divided by the radius that defines goodness, indicating that RED numbers in green (≤1) could be considered “good miscibility” between solvents and solutes whereas RED numbers in red (>1) correspond to a relatively bad miscibility. As can be seen in Figure 6, animal fats are theoretically proved to be preferred solvents for the extraction of carotenoids as compared to n-hexane and acetone, which have divergences with experimental results. However, the dissolving power among animal fat solvents represented higher predictive accuracy, where chicken fat performed the best for the extraction of carotenoids. For more polar capsaicinoids, all solvents showed the bad theoretical miscibility due to their high RED values whereas basa fish oil performed the best among animal fats in the real extraction of both capsaicin and dihydrocapsaicin. This phenomenon might be attributed to the formation of nano-scale lamellar structures stabilized by the monolayer of surfactant molecules like sterols, which contain hydrophilic cores that enable the coexistence of both polar and non-polar compounds in the animal fat system [7]. Overall, animal fats showed a better theoretical miscibility with both carotenoids and capsaicinoids than conventional petroleum-based volatile solvents according to their RED numbers whereas the real experiments presented the opposite results. Hence, it is important to note that the theoretical miscibility based on thermodynamics rather than kinetics could only help to estimate whether it is thermodynamically possible for a solvent to dissolve the solute or not, it cannot quantitatively decide the real solubility of solutes in solvents. However, the inconsistency with experimental results in case-by-case studies could help to improve the accuracy and robustness of HSPiP as the heuristic tool for a better comprehension of dissolving mechanisms for unconventional solvents.

## 4. Conclusions

Given the results above, the potential of using animal fats as food-grade solvents to simultaneously extract carotenoids and capsaicinoids from chili was verified, which is in conformity with Green Extraction principles in terms of using renewable raw materials and green solvents, reduced process unit and waste, energy savings and value-added final extracts. After oleo-extraction, there was no significant changes found in fatty acid composition, thermal behaviors and crystal polymorphism. The oxidative stability of oleo-extracts could be significantly improved by at least double and their acid values also increased but within an acceptable level. Based on both theoretical and experimental results, it can be concluded that animal fats show their different dissolving capacities depending on their types and compositions, where lard (60.39°) and basa fish oil (61.8°) after extraction could even reach a pungency degree comparable to commercial hotpot oil (66.76°). This may inspire a novel way of developing alternative Sichuan spicy hotpot oil with enhanced flavor and stability. Also, it is worth exploring how to improve this oleo-extraction ratio without quality degradations using innovative techniques (e.g., ultrasound, etc.) on the present basis in future investigations.

## Figures and Tables

**Table 1:** The total capsaicinoid content and pungency degree of animal fats after extraction.

Sample	Capsaicinoid Content (mg/g)	Pungency Degree (°)	Grade
Lard	0.63	60.39	Extra spicy
Beef tallow	0.59	59.75	High spicy
Chicken fat	0.58	59.54	High spicy
Basa fish oil	0.72	61.80	Extra spicy
Commercial hotpot oil	1.15	66.76	Extra spicy

**Table 2:** Fatty acid composition of different animal fats before and after extraction.

Fatty Acid Composition	Lard		Beef Tallow		Chicken Fat		Basa Fish Oil	
	Before	After	Before	After	Before	After	Before	After
C14:0	1.44 ± 0.04	1.40 ± 0.06	2.63 ± 0.16	2.59 ± 0.03	24.10 ± 0.00	24.10 ± 0.00	4.62 ± 1.15	3.72 ± 0.44
C14:1	/	/	0.50 ± 0.02	0.45 ± 0.05	/	/	/	/
C16:0	27.62 ± 0.24	27.31 ± 0.17	31.51 ± 1.13	30.92 ± 0.52	4.60 ± 0.00	4.47 ± 0.00	44.26 ± 0.06 ^g^	42.31 ± 0.44 ^h^
C16:1	1.42 ± 0.02	1.37 ± 0.10	3.41 ± 0.28	3.19 ± 0.04	/	/	/	/
C18:0	18.73 ± 0.51	18.07 ± 0.14	22.96 ± 0.98	21.98 ± 0.45	6.31 ± 0.00	6.27 ± 0.00	13.07 ± 0.33 ^i^	12.22 ± 0.08 ^j^
C18:1	35.67 ± 0.25	35.28 ± 0.38	35.74 ± 0.70 ^e^	34.33 ± 0.49 ^f^	37.02 ± 0.00	36.16 ± 0.00	29.89 ± 1.72 ^k^	30.85 ± 0.18 ^l^
C18:2	12.94 ± 0.10 ^a^	14.39 ± 0.16 ^b^	4.00 ± 0.15 ^c^	6.06 ± 0.07 ^d^	25.61 ± 0.00	26.66 ± 0.00	8.18 ± 0.22 ^m^	10.33 ± 0.07 ^n^
C18:3	/	/	/	/	2.11 ± 0.00	2.12 ± 0.00	0.87 ± 0.08	0.96 ± 0.01
SFA/UFA ratio	0.96	0.92	1.31	1.26	0.44	0.44	1.59	1.38

SFAs: saturated fatty acids; UFAs: unsaturated fatty acids. Values followed by the same letter are not significant at *p* < 0.05, which are presented as mean ± standard deviation of triplicate.

**Table 3:** Oil quality indexes of animal fats before and after extraction.

Index	Lard		Beef Tallow		Chicken Fat		Basa Fish Oil	
	Before	After	Before	After	Before	After	Before	After
Acid value (mg/g)	0.45 ± 0.03	1.58 ± 0.13 **	1.52 ± 0.00	2.78 ± 0.15 **	0.94 ± 0.11	1.54 ± 0.08 **	0.41 ± 0.05	1.16 ± 0.19 **
Peroxide value (meq O_2_/kg)	2.48 ± 0.5	1.16 ± 0.28 *	4.44 ± 0.24	0.98 ± 0.24 **	4.34 ± 0.51	2.36 ± 0.23 **	2.00 ± 0.35	0.68 ± 0.18 *
*p*-anisidine value	1.17 ± 0.06	0.51 ± 0.04 **	3.62 ± 0.56	1.31 ± 0.15 **	8.27 ± 0.30	2.90 ± 0.32 **	1.49 ± 0.28	0.40 ± 0.03 **
Total oxidation value (meq O_2_/kg)	6.12 ± 0.84	2.84 ± 0.47 **	17.09 ± 1.89	7.62 ± 0.89 **	12.36 ± 0.74	3.25 ± 0.28 **	4.89 ± 0.57	1.74 ± 0.38 **
Oxidation induction time (h)	3.61 ± 0.32	13.45 ± 0.54 **	18.89 ± 0.68	38.15 ± 6.44 **	2.03 ± 0.71	5.45 ± 0.70 *	19.67 ± 0.25	17.52 ± 0.22 **

Values followed by * and ** are significantly different at *p* < 0.05 and *p* < 0.01, respectively.

## Data Availability

The original contributions presented in the study are included in the article, further inquiries can be directed to the corresponding author.
